# Computationally efficient barycentric interpolation of large grain boundary octonion point sets

**DOI:** 10.1016/j.mex.2022.101731

**Published:** 2022-05-18

**Authors:** Sterling G. Baird, Eric R. Homer, David T. Fullwood, Oliver K. Johnson

**Affiliations:** Brigham Young University, USA

**Keywords:** Hypersphere, Octonion, Triangulation, Grain boundary

## Abstract

We present a method for performing efficient barycentric interpolation for large grain boundary octonion point sets which reside on the surface of a hypersphere. This method includes removal of degenerate dimensions via singular value decomposition (SVD) transformations and linear projections, determination of intersecting facets via nearest neighbor (NN) searches, and interpolation. This method is useful for hyperspherical point sets for applications such as grain boundaries structure-property models, robotics, and specialized neural networks. We provide a case study of the method applied to the 7-sphere. We provide 1-sphere and 2-sphere visualizations to illustrate important aspects of these dimension reduction and interpolation methods. A MATLAB implementation is available at github.com/sgbaird-5dof/interp.

• Barycentric interpolation is combined with hypersphere facet intersections, dimensionality reduction, and linear projections to reduce computational complexity without loss of information
• A max nearest neighbor threshold is used in conjunction with facet intersection determination to reduce computational runtime.

## Graphical abstract

**Figure:**
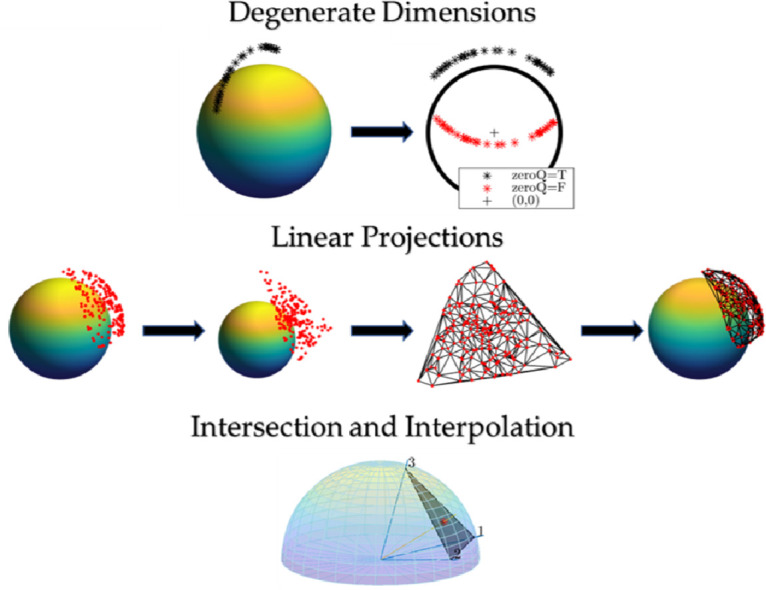

Specifications table

**Table:** 

Subject Area:	Materials Science
More specific subject area:	Barycentric Interpolation
Method name:	Approximate and Efficient N-sphere Barycentric Interpolation
Name and reference of original method:	Companion work (1) Baird, S. G.; Homer, E. R.; Fullwood, D. T.; Johnson, O. K. Five Degree-of-Freedom Property Interpolation of Arbitrary Grain Boundaries via Voronoi Fundamental Zone Framework. Computational Materials Science 2021, 200, 110756. https://doi.org/10.1016/j.commatsci.2021.110756. Singular value decomposition (2) Golub, G. H.; Reinsch, C. Singular Value Decomposition and Least Squares Solutions. Numerische Mathematik 1970, 14 (5), 403–420. https://doi.org/10.1007/BF02163027. Barycentric coordinates (3) Mobius, A. F.; Barth, J. A. Der Barycentrische Calcül Ein Neues Hülfsmittel Zur Analytischen Behandlung Der Geometrie; Johann Ambrosius Barth: Leipzig, 1827. Facet intersection (4) Anatoliy, T. Check if ray intersects internals of D-facet https://math.stackexchange.com/q/1256236. Quickhull Triangulation (5) Barber, C. B.; Dobkin, D. P.; Huhdanpaa, H. The Quickhull Algorithm for Convex Hulls. ACM Trans. Math. Softw. 1996, 22 (4), 469–483. https://doi.org/10.1145/235815.235821.Triangulation
Resource availability:	github.com/sgbaird-5dof/interp

*Method details

## Introduction

Barycentric coordinates are a type of homogeneous coordinate system that reference a prediction point within a simplex [1] or convex polytope [1], [2], [3] based on “masses” or weights at the vertices, which can be negative. The prediction point is assumed to be the barycenter (center of mass) of the simplex or convex polytope, and weights at the vertices necessary to make this assumption true are determined. We utilize rigid SVD transformations and a standard triangulation algorithm^1^ to define a simplicial mesh on the (approximated) surface of an n-dimensional hypersphere (Section 2). We then use barycentric weights (i.e. coordinates) for computing intersections of a point within a simplicial facet (Section 3) and for interpolation (Section 4) [1]. These methods are relevant for applications such as grain boundaries structure-property models [5], robotics hand-eye calibration [6], and efficient neural networks [7], especially cases where coordinates may have degenerate dimensions, occupy no more than a hemisphere^2^, or require interpolation of properties. While the methods described are general to n-dimensional hyperspheres, we focus on one application of interest for our prior work on grain boundary octonions [8]: the unit 7-sphere. Grain boundary octonions are of particular interest to us due to their ability to represent the macroscopic crystallographic character of grain boundaries and because they allow for an analytical minimum to the U(1) (z-axis) symmetry present in that representation which in other representations is solved by numerical methods (Section 2.1). This represents a significant advantage for computationally efficient computation of high-fidelity distance metrics between grain boundaries of varying macroscopic crystallographic character. Additionally, the novelty in our companion work ([8]) is the ability to compute distances between grain boundaries using Euclidean or hyperspherical distances in such a way that approximates the original grain boundary octonion distance metric [5,9]. This lends itself to Barycentric interpolation (this work). For further information on barycentric coordinates and its applications and generalizations, see [[1], [2], [3], [10], [11], [12], [13], [14], [15], [16], [17], [18], [19], [20], [21], [22], [23]]. The methods described here are used in Baird et al. [8] and are summarized in Table 1.

**Table 1:** Steps for mesh triangulation, mesh intersection, and barycentric interpolation for computationally efficient interpolation of properties on an n-sphere using large point sets.

Step	(1) Mesh Triangulation
1.1	apply a SVD transformation to remove any degeneracies (Section 2.1)
1.2	project to tangent hyperplane relative to origin and mean of input points (Section 2.2)
1.3	perform a second SVD transformation (Section 2.3)
1.4	compute the triangulation according to the quickhull algorithm [4]

Step	(2) Mesh Intersections

2.1	apply the same rigid transformation to the prediction points (Section 3.1)
2.1a	concatenate both input and prediction points
2.1b	perform the SVD transformation
2.1c	separate the transformed input and prediction points (reverse of concatenation step)

2.2	identify facets nearby a prediction point and test for intersection (Section 3.2)
2.2a	linearly project the prediction point onto facet hyperplane
2.2b	compute the point's barycentric coordinates within the facet [21,24]
2.2c	test that all coordinates are positive [1] within a tolerance
2.2d	repeat steps 2.2a-2.2c until an intersection is found or a stop condition is reached

Step	(3) Barycentric Interpolation

3.1	Recompute barycentric coordinates using a larger tolerance
3.2	Compute dot product between barycentric coordinates facet vertex properties

## Triangulating a mesh

Creation of a simplicial mesh is necessary to perform barycentric interpolation. Due to the difficulty of interpreting and visualizing a high-dimensional n-sphere [9], we provide visual illustrations of the process as applied to lower-dimensional analogues. The triangulation process occurs by:

1.1 applying a SVD transformation to remove any degeneracies^3^ (Section 2.1)
1.2 linearly projecting points onto a hyperplane that is tangent to the vector between the origin and the mean of the input points^4^ (Section 2.2)
1.3 performing a second SVD transformation (Section 2.3)
1.4 computing the triangulation according to the quickhull algorithm [4]

**Fig. 1:**
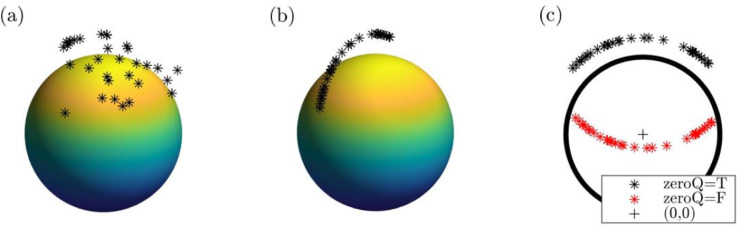
3D Cartesian to 2D Cartesian analogue of 8D Cartesian to 7D Cartesian degeneracy removal via rigid SVD transformation as used in barycentric interpolation approach. (a) Starting spherical arc points on surface of 2-sphere, (b) rotational symmetrization applied w.r.t. z-axis (analogous to U(1) symmetrization), and (c) degenerate dimension removed via singular value decomposition transformation to 2D Cartesian with either the origin (black plus) preserved (black asterisks, zeroQ=T) for triangulation or ignored (red asterisks, zeroQ=F) for mesh intersection. The spheres (a,b) and circle (c) each have a radius of 0.8 and are used as a visualization aid only.

**Fig. 2:**
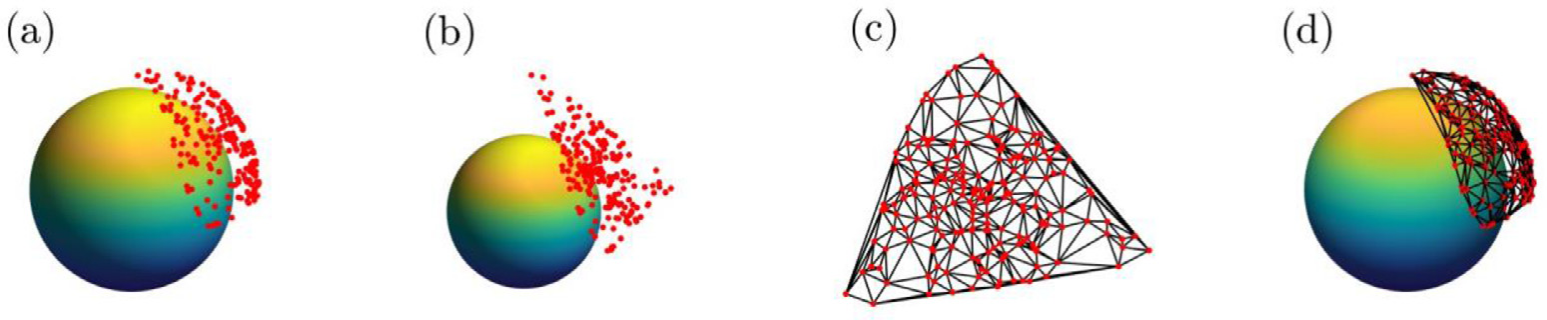
3D Cartesian to 2D Cartesian analogue of 7D Cartesian to 6D Cartesian mesh triangulation used in barycentric interpolation approach. (a) 3D Cartesian input points are (b) linearly projected onto hyperplane that is tangent to mean of starting points. (c) The degenerate dimension is removed via a rigid SVD transformation to 2D Cartesian and the Delaunay triangulation (black lines) is calculated, with input vertices (red). Delaunay triangulation superimposed onto normalized input points (d). The spheres in (a), (b), and (d) have a radius of 0.8 and are used for visualization only.

**Fig. 3:**
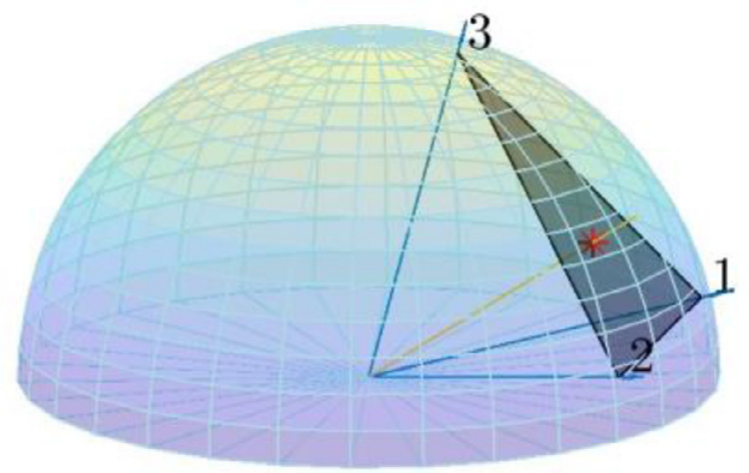
A ray (red line) is linearly projected from the 2-sphere onto the hyperplane of a mesh facet (transparent black), shown as a red asterisk. The barycentric coordinates are computed as. Because all barycentric coordinates are positive, it is determined that the projected point is an intersection with the mesh. Given vertex values of 8*.*183, 3*.*446, and 3*.*188 for vertices 1, 2, and 3, respectively, the interpolated value is calculated as 4*.*94 via Eq. (5).

In the explanation of each step that follows, we make reference to lower-dimensional visual analogues of the triangulation procedure, which are given in Figs. 1–3. We note that 3D Cartesian coordinates in Fig. 1 correspond to 8D Cartesian coordinates, whereas 3D Cartesian coordinates in Figs. 2 and 3 correspond to 7D Cartesian coordinates. This is intentional for two reasons.

First, Fig. 1 illustrates that 8D Cartesian points constrained to the surface of a hypersphere are analogous to a point cloud on the 2-sphere (Fig. 1a) and that an 8D Cartesian point set constrained to the surface of a hypersphere is analogous to a geodesic arc on the 2-sphere (Fig. 1b). If a point set has a degenerate dimension, this can be removed by a rigid SVD transformation to 7D Cartesian coordinates (analogous to 2D Cartesian coordinates in Fig. 1c). This sequence would be more difficult to visualize if Fig. 1a was meant to represent a point cloud on the 3-sphere (4D Cartesian coordinates), etc.

Second, Fig. 2 illustrates a second transformation from normalized 7D Cartesian coordinates (Fig. 2a) to a hyperplane (Fig. 2b) which is then transformed into 6D Cartesian coordinates via a second SVD. In this case, key issues are retained that would otherwise be lost if an arc on a circle (1-sphere) to 1D Cartesian coordinates were used instead^5^. Additionally, the use of actual triangles is a more familiar and compelling illustration of *triangulation*.

While lower dimensional analogues are useful for visualizing and understanding the process of triangulation, a written description for the full-dimensional space is also given (Sections 2.1–2.3). As appropriate, we refer to the teaching figures described in this section.

### Singular value decomposition transformation from 8D Cartesian to 7D Cartesian

To reduce the computational complexity of triangulating a high-dimensional mesh [4], some simplifications are made. First, the degenerate dimension which is present from the analytically minimizing *U*(1) symmetry [9] is removed via a rigid (i.e. distance- and angle-preserving) SVD transformation. This is analogous to a Cartesian rotation and translation (see 3D to 2D SVD transformation from Fig. 1b to c). A SVD is given by:

(1) A=USV′

where *U, S, V*, and ·′ represent a unitary matrix with sorted, singular vectors, a diagonal matrix containing sorted, singular values, a unitary matrix with sorted, singular vectors, and Hermitian transpose operator, respectively. The SVD transformed coordinates [24] are given by:

(2) $$\overset{B}{\overset{︷}{\left(\begin{array}{cccc} b_{1,1} & b_{1,2} & \ldots & b_{1,\left(n-\text{nforce}\right)}\\ b_{2,1} & b_{2,2} & \ldots & b_{2,\left(n-\text{nforce}\right)}\\ \;\;\vdots & \;\;\vdots & \ddots & \;\;\;\;\vdots \\ b_{m,1} & b_{m,2} & \ldots & b_{m,\left(n-\text{nforce}\right)} \end{array}\right)}}=\overset{U}{\overset{︷}{\left(\begin{array}{cccc} u_{1,1} & u_{1,2} & \ldots & u_{1,n}\\ u_{2,1} & u_{2,2} & \ldots & u_{2,n}\\ \;\;\vdots & \;\;\vdots & \ddots & \;\;\;\;\vdots \\ u_{m,1} & u_{m,2} & \ldots & u_{m,n} \end{array}\right)}}\overset{S_{sub}}{\overset{︷}{\left(\begin{array}{cccc} s_{1,1} & s_{1,2} & \ldots & s_{1,\left(n-\text{nforce}\right)}\\ s_{2,1} & s_{2,2} & \ldots & s_{2,\left(n-\text{nforce}\right)}\\ \;\;\vdots & \;\;\vdots & \ddots & \;\;\;\;\vdots \\ s_{n,1} & s_{n,2} & \ldots & s_{n,\left(n-\text{nforce}\right)} \end{array}\right)}}$$

where *n*_deg_ and *S*_sub_ represent number of degenerate dimensions and *S* with the degenerate columns removed, respectively. Only the principal components which correspond to non-degenerate dimensions are retained (this process of dimensionality reduction is also referred to as principal component analysis).

### Linearly project onto hyperplane

Next, the resulting 7D Cartesian representation of each point is projected onto a hyperplane that is tangent to the centroid (i.e. mean) of the point set^6^ (Fig. 2a). The linear projection is given by [24]:

(3) $$a=-\frac{∥v∥}{v\cdot p}p$$

where v, p, kk, and • represent unit normal to hyperplane of interest, point to project from hypersphere to hyperplane, 2-norm, and dot product, respectively. By performing this linear projection, one of the dimensions becomes degenerate.

### Singular value decomposition transformation from 7D Cartesian to 6D Cartesian

This additional degeneracy is removed via a second SVD transformation, this time to 6D Cartesian coordinates (see 3D to 2D projection in Fig. 2a-b).

The distortion-introducing local SVD is simply to efficiently obtain a triangulation which then applies directly on the (one-dimension higher) point set, which is where interpolation occurs. Since the distortion is isotropic with respect to solid angle, we believe the triangulation is very similar to what would be obtained without SVD and that the interpolation will be largely unaffected, and this is what we see in practice.

Stereographic projections are an alternative method to SVD that could have been used; there do not seem to be clear methodology advantages of using one or the other for our use case of grain boundary octonions; because well-maintained software implementations for SVD are more prevalent, we determined this to be the more favorable choice.

Finally, the resulting points can be triangulated via the quickhull algorithm [4]^7^ which relies on Euclidean distances^8^. Because the simplicial mesh is defined by a list of edges between vertices for each simplicial facet, this list applies immediately to the point set in its 7D Cartesian coordinates (i.e. no reverse transformation is necessary to use the mesh on the 6-sphere in 7D).

## Intersections in a mesh

Once the triangulation has been determined, we need to find which facet each prediction point intersects (i.e. find the intersecting facet). There are two sub-steps:

2.1 apply the same rigid transformation to the prediction points as was applied to the input points (otherwise the prediction points will not line up properly with the mesh) (Section 3.1)
2.2 identify facets nearby a prediction point and test for intersection (Section 3.2).

### Apply same singular value decomposition to input and prediction points

The positions of the prediction points need to be fixed relative to the mesh even after the rigid SVD transformation. This is accomplished by:

2.1a concatenating both input and prediction points
2.1b performing the SVD transformation^9^
2.1c subsequently separating the transformed input and prediction points (reverse of concatenation step)

The same SVD transformation can be applied without major issue to new points, assuming the new points are not positioned outside the bounds of the original convex hull^10^.

### Testing nearby facets for intersections

Once the prediction points are lined up properly with the mesh, the facet containing the prediction point (i.e. intersecting facet) is found^11^. We define the intersecting facet as the one for which a point's barycentric coordinates are positive within a given tolerance:

(4) $$\lambda_{i}\geq -\sigma ,\;i\in \left[1..d\right]$$

where *λ_i_, σ*, and d represent i-th barycentric coordinate, projection (or intersection) tolerance, and dimension of barycentric coordinates, respectively. Consequently, we determine facet affiliation by:

2.2a linearly projecting the prediction point onto the hyperplane defined by a mesh facet's vertices (Fig. 3)
2.2b computing^12^ the point's barycentric coordinates within the facet [21,24]
2.2c testing that all coordinates are positive [1] within a tolerance. Two tolerances^13^ are used: one for the initial computation of barycentric coordinates by projecting onto the hypersphere to determine facet affiliation and a larger tolerance for computation of barycentric coordinates to determine interpolated values (Section 4).
2.2d repeating steps 2.2a-2.2c until an intersection is found or a stop condition is reached^14^

**Fig. 4:**
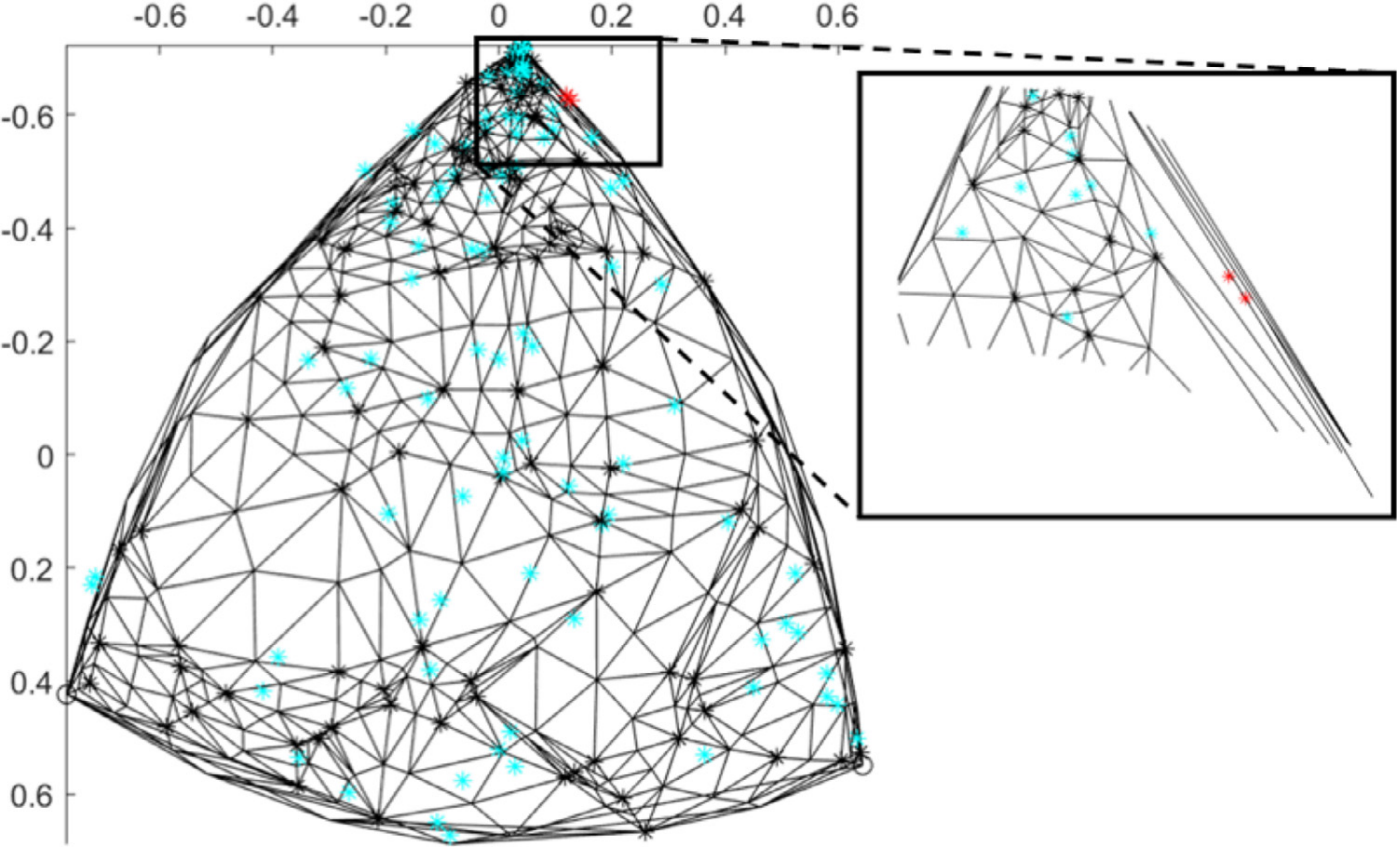
Illustration of two prediction points (red) for which no intersecting facet is found due to being positioned within a high-aspect ratio facet. The inset shows that facets connected to the NN do not contain the prediction point. Many NNs would need to be considered before an intersection is found. Additionally, it is expected that if found, the interpolation will suffer from higher error due to use of facet vertices far from the interpolation point. Proper intersections of prediction points with the mesh are shown in blue.

Due to the large number of facets per point of a high-dimensional triangulation (approximately 2000 facets per vertex for a 50000 point triangulation, or 1 × 10^8^ total facets), some simplifications are made in order to determine intersections of prediction points with the mesh. If every edge length of every facet were equal, only facets connected to the first NN would need to be considered to find a proper intersection. However, since the points are randomly sampled, edge lengths of facets are non-uniform, and non-unity aspect-ratio facets exist (Fig. 2, Fig. 4). If the facets have high-aspect ratios, the intersecting facets of prediction points can be far from the NNs mesh points relative to the prediction points (see Fig. 4 inset), especially near the perimeter of a hyperspherical surface mesh. Rather than loop through every facet to find an intersection (∼1 × 10^8^ facets in a 50000 point mesh), the prediction point intersections are calculated by considering facets connected to up to some number of NN mesh vertices^16^ relative to each prediction point. The NN mesh vertices relative to a prediction point are computed. The facet IDs of facets connected to these NNs are then computed^15^.

Some prediction points will have no intersecting facet found. From our numerical testing, we determine that this non-intersection phenomenon occurs in two situations:

• high-aspect ratio facets (described above)
• prediction points that are positioned just outside the bounds of the mesh but within the bounds of a region, due to the fact that the mesh is a piecewise linear approximation of a surface with a curved perimeter and that randomly sampled points typically do not fall on the true perimeter

In the first case, barycentric interpolation within high-aspect ratio facets may actually lead to worse interpolation error than a NN interpolation strategy due to influence by points far from the prediction point. In the second case, there is no true intersection between the prediction point and the mesh. Both issues can be addressed with the same strategy: we apply a NN approach when an intersecting facet is not found within some number of NNs. In numerical tests, meshes composed of 388 and 50000 vertices produced non-intersection rates of (12*.*07 ± 1*.*02)% and (0*.*68 ± 0*.*11)%, respectively, over approximately 10 trials and using 10000 prediction points for each trial. An alternative strategy for the second case is to linearly project the point of interest onto the closest facet, and compute the interpolation there.

## Interpolation

Once a mesh triangulation has been determined (Section 2), barycentric coordinates are recomputed for a prediction point within the input mesh (Section 3) using a somewhat larger tolerance; the interpolated value is found by taking the dot product of the prediction point's barycentric coordinates and the properties of the corresponding vertices of the intersecting facet via

(5) $$v_{m,q}=\sum_{i=1}^{N}\lambda_{m,i}v_{m,i}$$

where *λ_m,i_, v_m,q_, v_m,i_* and *N*, are the barycentric coordinates of the m-th prediction point, interpolated property at the m-th prediction point, property of the *i*-th vertex of the intersecting facet for the mth prediction point, and number of vertices in a given facet (*N* = 7 for facets of the simplicial mesh on the degeneracy-free 6-sphere), respectively. Interpolation of many prediction points simultaneously can be accomplished by a simple, vectorized approach^16^. This assumes triangulation and weights have been precomputed. In other words, both input and prediction coordinates remain fixed, and only input property values change. If this is the case, barycentric interpolation of new points is incredibly fast. By contrast, if input coordinates change, the triangulation must be recomputed, and if prediction coordinates change, the intersecting facets must be recomputed.

In Baird et al. [8], we also performed barycentric interpolation without the centroid projection described (thereby removing the local distortion inherent to the local SVD transformation) and found only insignificant differences for our application. For interested readers, the implementation for the more exact spherical Barycentric method is also available at https://github.com/sgbaird-5dof/interp.

## Efficiency

Both triangulation and finding intersecting facets are computationally demanding with respect to memory and runtime for large datasets. A mesh triangulation consisting of 50000 points evaluated for 10000 interpolation points requires ∼1.6 hours with 12 cores (∼20 CPU hours in total) and 128 GB of RAM available. The total runtime as a function of set size evaluated on 10000 prediction points (i.e. combined triangulation and intersection finding) is estimated by a fitted linear model^17^, 5332*.*02 +

1*.*26959*x*, where *x* is the number of points and 1000 ≤ *x* ≤ 50000. The triangulation itself (∼1 × 10^8^ facets) requires ∼6 GB of memory storage. Alternative interpolation methods such as Gaussian process regression and other machine learning approaches can greatly reduce the computational burden while retaining interpolative performance [8].

## Future work

It may be interesting to compare these Barycentric interpolation techniques (general to n-spheres) with techniques that leverage unique algebra specific to hypersphere dimensions such as the 1-, 3-, 7-, and 23-spheres. For example, is using one of these explicit parameterizations faster, and will this be feasible when degeneracies specific to grain boundary octonions are present?

## Conclusion

SVD/PCA transformations, linear projections, and nearest neighbor searches can be used to reduce the computational burden of high-dimensional hyperspherical triangulations and intersection-finding. Large point sets in high-dimensions still have large memory and runtime requirements, but are more tractable with these methods. When the borders of the region of interest are not within the convex hull of points or when the region of interest is inherently curved (beyond the curvature naturally present due to residing on a hypersphere), non-intersections manifest and can be addressed by defaulting to a nearest neighbor approach.

## Declaration of Competing Interests

The authors declare that they have no known competing financial interests or personal relationships that could have appeared to influence the work reported in this paper.

## Footnotes

1 i.e. quickhull [4] via delaunayn in sphconvhulln.m. Built-in MATLAB functions are indicated with parentheses (e.g. delaunayn), whereas custom functions (from github.com/sgbaird-5dof/interp unless otherwise specified) are indicated with the .m extension (e.g. sphconvhulln.m).

2 When the points occupy less less than a hemisphere, a linear projection onto a hyperplane followed by an SVD transformation can be used to compute the triangulation in one dimension lower^3^ If the points span the full hypersphere, triangulation must proceed in the original dimensionality to produce an accurate triangulation.

5 Non-intersection issues due to high-aspect ratios and consideration of facets connected up to nnMax NNs do not manifest in triangulations on the surface of a 1-sphere because one of the two facets (i.e. line segments) connected to the first NN mesh vertex relative to the prediction point is guaranteed to have an intersection.

6 This is *not* a rigid transformation; however, it approximates one with sufficient accuracy to produce a high-quality triangulation.

7 See sphconvhulln.m and delaunayn.

8 While the triangulation algorithm used in this work relies on Euclidean distances, other distance metrics that are non-Euclidean [25] could potentially be incorporated into the barycentric approach such as by doing an edge-length based simplex reconstruction [26,27] using the triangulation edge lengths.

10 To map new points onto the mesh, the usv structure output from proj_down.m needs to be stored and supplied in future calls to proj_down.m. Likewise, usv need to be supplied to proj_up.m to perform the reverse SVD transformation.

11 Testing intersections for nearby facets is handled in intersect_facet.m and depends on the barycentric coordinate computations in projray2hypersphere.m.

15 via find(K==nn), where K is the triangulation from sphconvhulln.m and nn is the ID of one of the NN mesh vertices)

16 i.e. dot as used in interp_bary_fast.m.

17 Using Mathematica's FindFormula.

## Footnotes

3 e.g. U(1)-symmetry degeneracy for grain boundary octonions [9]) inherent in the coordinates.

4 This applies when the data resides on less than a hemisphere and is to reduce computational burden of the triangulation.

9 See proj_down.m via svd.

12 See projray2hypersphere.m

13 We typically use projtol=1e-4 in proj_down.m and inttol=1e-2 in intersect_facet.m, respectively.

14 The stop condition is that up to a certain number of NN have been considered (or all points have been considered). The parameter associated with this is nnMax in intersect_facet.m ^16^See nnMax in intersect_facet.m. We have typically used nnMax=10.
